# Bedtime procrastination: introducing a new area of procrastination

**DOI:** 10.3389/fpsyg.2014.00611

**Published:** 2014-06-19

**Authors:** Floor M. Kroese, Denise T. D. De Ridder, Catharine Evers, Marieke A. Adriaanse

**Affiliations:** Department of Clinical and Health Psychology, Utrecht UniversityUtrecht, Netherlands

**Keywords:** procrastination, self-regulation, sleep, health, MTurk

## Abstract

**Background**: Procrastination is a prevalent and problematic phenomenon that has mostly been studied in the domain of academic behavior. The current study shows that procrastination may also lead to harmful outcomes in the area of health behavior, introducing *bedtime procrastination* as an important factor related to getting insufficient sleep and consequently affecting individual well-being. Bedtime procrastination is defined as failing to go to bed at the intended time, while no external circumstances prevent a person from doing so.

**Methods**: To empirically support the conceptual introduction of bedtime procrastination, an online survey study was conducted among a community sample (*N* = 177). The relationship between bedtime procrastination and individual difference variables related to self-regulation and general procrastination was assessed. Moreover, it was investigated whether bedtime procrastination was a predictor of self-reported sleep outcomes (experienced insufficient sleep, hours of sleep, fatigue during the day).

**Results**: Bedtime procrastination was negatively associated with self-regulation: people who scored lower on self-regulation variables reported more bedtime procrastination. Moreover, self-reported bedtime procrastination was related to general reports of insufficient sleep above and beyond demographics and self-regulation.

**Conclusions**: Introducing a novel domain in which procrastinators experience problems, bedtime procrastination appears to be a prevalent and relevant issue that is associated with getting insufficient sleep.

## Introduction

Procrastination is a prevalent and problematic phenomenon. Its prevalence comes about in impressive figures showing, for example, that up to 46% of college students report to procrastinate on specific academic tasks (Solomon and Rothblum, 1984) and that about 10% of adults in the general population indicate to be chronic procrastinators (Ferrari et al., 2005). The notion that procrastination is problematic, then, is supported by studies showing that procrastinators typically perform more poorly in various domains including academic achievements and work activities, but also experience financial drawbacks due to procrastinating on taxes or failing to start retirement savings (Steel, 2007). The current study aims to highlight another domain in which procrastination may lead to harmful outcomes, introducing *bedtime procrastination* as an important factor related to getting insufficient sleep and consequently affecting individual well-being.

Procrastination is defined as a “voluntary delay of an intended course of action despite expecting to be worse off for the delay” (Steel, 2007, p. 66). Being reflective of self-regulatory failure, procrastination as a personality trait has clear associations with conscientiousness (reverse), impulsiveness, and low self-control (Steel, 2007). Although procrastination is now typically considered a general trait that manifests itself in various domains (Lay, 1986; Milgram et al., 1998), it has mostly been studied with regard to work and academic behavior (Van Eerde, 2003). This has led researchers to repeatedly call for procrastination research in other domains (e.g., Sirois et al., 2003; Van Eerde, 2003).

One imperative domain in which procrastination has received remarkably little attention is health behavior. Although it has been noted that, for example, procrastinators tend to experience higher stress and poorer health (Tice and Baumeister, 1997), less is known about the association between procrastination and the performance of health behaviors. A notable exception in this regard is the work by Sirois and colleagues (Sirois et al., 2003; Sirois, 2007) who, besides replicating findings that procrastination is associated with poorer health, higher stress, and greater treatment delay, showed that procrastinators practiced fewer wellness behaviors (e.g., lower fruit and vegetable intake, less physical activity). This is an important first indication that procrastination is not only the work-related performance problem as we all know it, but also transfers to other important life domains including health behavior.

What is as of yet unclear, however, is to what extent the association between procrastination and health behaviors could have been explained by the underlying factor of trait self-control. That is, trait self-control—the ability to override or change one's inner responses (Baumeister, 2002)—is known to be associated with both procrastination and health behaviors (Tangney et al., 2004; De Ridder et al., 2012), leaving open the possibility that the relationship between procrastination and health behaviors is merely reflective of a confounding general self-control problem rather than actually involving putting things off.

The current study will investigate a form of procrastination in a specific health domain: sleeping behavior, or more specifically, going to bed. Getting sufficient sleep is increasingly being recognized as essential for people to function in an optimal way, with studies showing that sleep deficiency is related to concentration and memory problems (Ram et al., 2010), but also to more severe outcomes such as obesity, hypertension, and cardiovascular disease (Buxton and Marcelli, 2010; Sabanayagam and Shankar, 2010). In this light it is striking that 28% of U.S. adults report to sleep only 6 h per night or less (Krueger and Friedman, 2009), while 7–9 h is conventionally considered appropriate. Although most research has been concerned with external factors or sleeping disorders as a cause for insufficient sleep, we propose that for many, the hours of sleep may be limited “simply” due to going to bed late. Different from many other health behaviors that are either or not performed (e.g., you either eat an apple or you don't), going to bed is more a matter of “when” rather than “if.” As such, going to bed is a typical health behavior that can be postponed (i.e., that people can procrastinate on). Anecdotal evidence indeed learns that *bedtime procrastination* is a common experience for many, but scientific literature describing this phenomenon is lacking.

Our first aim is to demonstrate that bedtime procrastination—going to bed later than intended while no external circumstances are accountable for doing so—is associated with general procrastination and self-regulation. This attests to our notion that going to bed later than intended is indeed a matter of procrastination, and that individuals who are procrastinators or poor self-regulators in general are also more likely to procrastinate on their bedtimes.

The present study includes several measures of self-regulation. First, we are interested in trait self-control, which is a dominant construct in self-regulation research (De Ridder et al., 2012) that has been consistently (negatively) linked to self-regulatory failure (Tangney et al., 2004). Conceptually, procrastination (i.e., not doing what one ought to do at a given moment) is a clear illustration of low self-control as well (Steel, 2007). We therefore expect a negative relation between trait self-control and bedtime procrastination. Second, we will take into account conscientiousness and impulsivity. Conscientiousness reflects the extent to which people are organized and responsible, and is the personality trait that is most consistently (negatively) associated with procrastination (Steel, 2007). Impulsivity, on the other hand, refers to the tendency to act without thinking (Baumeister, 2002). Thus, people who score low on conscientiousness or high on impulsivity would be expected to display less goal-consistent behavior and be more likely to experience bedtime procrastination. Finally, we include a measure of action control (Kuhl, 1994), distinguishing between action and state orientation. Whereas the former is associated with adaptive decision making and goal-directed behavior in difficult situations, people adhering to a state orientation tend to be more hesitant and likely to postpone action. Therefore, our prediction is to find a negative relation between action orientation and bedtime procrastination.

The second aim of the current paper, then, is to show that bedtime procrastination is associated with self-reported objective and subjective sleep outcomes (i.e., hours of sleep, daytime fatigue, and experienced sufficient sleep), above and beyond demographics and self-regulation. In this way, our study is the first to show an association between procrastination and a specific health behavior, which cannot be explained by an underlying factor like trait self-control. Importantly, our specific focus is on the normal population who have no actual constraints for going to bed in time, but “just” fail to do so. Hence, we exclude people who have been treated for sleeping disorders or who work night shifts, as these circumstances may have large impacts on getting insufficient sleep outside of a person's control.

In sum, the current paper contributes to the literature in two important ways: First, it puts forward a novel health behavior area in which procrastinators may experience problems, namely getting sufficient sleep. Second, the current paper contributes to understanding insufficient sleep as a personality-related problem that may require novel approaches in terms of potential solutions.

## Methods

### Participants

Participants were recruited through Amazon Mechanical Turk (www.MTurk.com), which is an online “marketplace for work” that can be used for research. MTurk workers are typically more diverse than usual internet samples, and they have been shown to yield high-quality data for psychological research (Buhrmester et al., 2011). Two hundred and three participants completed the survey. Data from seven participants who indicated to suffer from sleeping disorders and another 19 who reported to work nightshifts were excluded from analyses. Of the remaining sample (*N* = 177) 48.6% was male, and the mean age was 37.9 years (*SD* = 11.0). The majority, 72.3%, was employed (26% part-time). 55.4% was married or in a domestic partnership, and 35.6% was single (the remaining 9.0% was either widowed, divorced or separated). Regarding ethnicity, 70.1% were Caucasian, 19.8% Asian or Pacific Islander, 4.5% African American, and 4.5% Hispanic or Latino (1.1% other). Education level was diverse: 16.4% completed high school or less, 19.8% completed vocational education, 48.0% had a Bachelor's degree, and 15.8% a Master's or higher.

### Procedure

The survey was posted on the MTurk platform, and made available only to workers who had successfully participated in previous tasks with a 98% approval rate, to ensure high-quality data. The approval of completed tasks is given by the task requester (e.g., experimenter) based on accuracy and reliability judgments of the given responses. Participants earned $1 for completion of the 20-min survey.

The study was conducted in accordance with the ethical standards described by the Medical Research Involving Human Subjects Act (WMO, 2012). This Act exempts research on healthy human subjects from review for as long as it does not involve any invasion of participants' integrity. According to Dutch national guidelines our study was not invasive of participants' integrity, and hence not subject to the WMO. Informed consent was obtained from each participant prior to participation.

### Questionnaire

#### Demographics

Demographics included sex, age, marital status (single, married or domestic partnership, widowed, divorced, separated), ethnicity (Caucasian, Hispanic or Latino, African American, Native American, Asian or Pacific Islander, Other), education (elementary school, secondary school, vocational education, Bachelor's, Master's, Doctoral, or Professional degree), and employment status (employed fulltime, employed part time, unemployed and looking for work, unemployed and not looking for work, student, homemaker, retired, other). Finally, participants indicated the number of children under the age of 5 living in the same household.

#### Bedtime procrastination

A 9-item scale was developed to assess bedtime procrastination (Cronbach's α = 0.92; see Appendix). Items were answered on 5-point scales ranging from 1 *(never)* to 5 *(always)*. An exploratory factor analysis using principle component analysis revealed a single-factor solution (Eigenvalue = 5.57), indicating that the scale assesses a uniform construct, as intended.

#### General procrastination

General procrastination was assessed with the 20-item general procrastination scale (Lay, 1986; e.g., “I generally delay before starting on work I have to do,” Cronbach's α = 0.93). Answers were given from 1 (very uncharacteristic) to 5 (very characteristic). The scale was included to test the correlation between general procrastination and the specific domain of bedtime procrastination.

#### Sleep outcomes

*Hours of sleep* was assessed by asking “on average, how many hours a night do you sleep during weekdays,” which was answered on a 7-point scale ranging from “*less than 5 h*” with increments of 1 h (*5–6 h, 6–7 h*, …) to “*more than 10 h*.” *Daytime fatigue* was assessed with a single item: “on average, how many days a week do you feel tired during the day.” Finally, *experienced sleep insufficiency* was assessed with: “on average, how many days a week do you feel you have slept too little.” The latter two questions were answered on 5-point scales (*0, almost never; 1–2 days; 3–4 days; 5–6 days; 7 days, almost always*).

#### Self-regulation

Four individual difference variables associated with self-regulation were assessed. *Self-control* was assessed with the 13-item brief self-control scale (Tangney et al., 2004; e.g., “I am good at resisting temptations,” Cronbach's α = 0.86) with answers from 1 *(not at all like me)* to 5 *(just like me)*. *Conscientiousness* was assessed with the 9-item subscale of the Big Five Inventory (John et al., 1991, 2008). Items (e.g., “I am someone who does a thorough job,” Cronbach's α = 0.86) were rated from 1 *(strongly disagree)* to 5 *(strongly agree)*. *Impulsivity* was assessed with the short form of the Barratt Impulsivity Scale (BIS-15; Spinella, 2007). The 15 items (e.g., “I plan tasks carefully,” reverse coded; Cronbach's α = 0.85) were answered on a scale from 1 *(rarely)* to 4 *(almost always)*. *Action control* was assessed with the Action Control Scale (ACS-24; Kuhl and Beckmann, 1994). For the current purpose, we were specifically interested in the decision subscale (AOD), consisting of 12 items that each have 2 alternative answers corresponding to a state or an action orientation (e.g., “When I know I must finish something soon, (A) I have to push myself to get started; (B) I find it easy to get it done and over with”). For the analyses, a sum of the action-oriented answers was created.

## Results

### Descriptives

Table 1 presents the descriptive statistics of the sleep outcome variables. Interestingly, almost 30% of the sample reported to sleep only 6 h or less on week nights. Additionally, about 84% reported to feel having slept too little or to feel tired during the day at least once a week, with more than 40% reporting this for 3–4 days or more. These figures confirm the importance of studying insufficient sleep in general population samples. Furthermore, participants on average reported moderate levels of bedtime procrastination (*M* = 2.8, *SD* = 0.8), indicating that the phenomenon is indeed commonly experienced.

**Table 1:** **Descriptive statistics of sleep outcome variables**.

**(A) FREQUENCIES OF SLEEP HOURS**						
	**On average, how many hours a night do you sleep during the week?**					
		**Frequency**		**%**		**Cum. %**
Less than 5 h		4		2.3		2.3
5–6 h		48		27.1		29.4
6–7 h		60		33.9		63.3
7–8 h		45		25.4		88.7
8–9 h		16		9.0		97.7
9–10 h		2		1.1		98.9
More than 10 h		2		1.1		100.0
**(B) FREQUENCIES OF SLEEP OUTCOMES**						
	**How many days a week do you feel you have slept too little?**			**How many days a week do you feel tired during the day?**		
	**Frequency**	**%**	**Cum. %**	**Frequency**	**%**	**Cum. %**
0	29	16.4	16.4	26	14.7	14.7
1–2 days	79	44.6	61.0	78	44.1	58.8
3–4 days	46	26.0	87.0	45	25.4	84.2
5–6 days	17	9.6	96.6	16	9.0	93.2
7 days	6	3.4	100.0	12	6.8	100.0

### Associations between key variables

For self-regulation, a compound measure was created as the individual variables were highly correlated (ranging from *r* = 0.55 to 0.73; cf. Duckworth and Seligman, 2005). First, impulsivity items were recoded such that higher scores reflected lower impulsivity (i.e., higher self-control), in accordance with the interpretation of the self-control, conscientiousness, and action orientation scales. Then, a mean was computed of standardized scores on each scale. On the composite measure, higher scores reflect better self-regulation. Correlations between the composite measure and the four individual variables ranged from 0.82 to 0.89.

Table 2 presents the zero-order correlations between demographics, bedtime procrastination, general procrastination, self-regulation and sleep outcomes. As predicted, bedtime procrastination was negatively associated with self-regulation. Notably, the correlation between bedtime procrastination and general procrastination (*r* = 0.60, *p* < 0.001) reflects considerable overlap but also distinctiveness of the sleep-specific type of procrastination and the general trait. Furthermore, confirming the validity of the context-specific bedtime procrastination scale, it was found that the associations with sleep outcomes were much higher for bedtime procrastination (ranging from *r* = 0.46 to 0.61) than for general procrastination (ranging from *r* = −0.19 to 0.35)^1^.

**Table 2:** **Zero-order correlations**.

	**1**	**2**	**3**	**4**	**5**	**6**	**7**	**8**	**9**	**10**
1. Gender										
2. Age	0.11									
3. Marital status (dummy)	0.15^*^	0.19^*^								
4. Employment (dummy)	−0.02	−0.02	−0.02							
5. Children < age 5	0.08	−0.16^*^	0.36^*^	0.04						
6. Bedtime procrastination	0.07	−0.11	0.01	−0.04	0.09					
7. General procrastination	0.08	−0.18^*^	−0.03	−0.05	0.06	0.60^*^	–			
8. Self-regulation	−0.04	0.14	0.05	0.12	−0.01	−0.52^*^	−0.81^*^	–		
9. Hours of sleep	0.02	0.13	−0.02	−0.10	−0.12	−0.49^*^	−0.19^*^	0.20^*^	–	
10. Fatigue	−0.01	−0.14	−0.08	−0.19^*^	−0.00	0.46^*^	0.37^*^	−0.40^*^	−0.29^*^	–
11. Insufficient sleep	−0.04	−0.17^*^	0.04	−0.04	0.14	0.61^*^	0.35^*^	−0.39^*^	−0.54^*^	0.65^*^

* Correlations significant at p < 0.05.

### Bedtime procrastination as a predictor of sleep outcomes

Three hierarchical regression analyses tested the effect of bedtime procrastination on (a) reported hours of sleep, (b) daytime fatigue, and (c) experienced insufficient sleep, above and beyond demographics and self-regulation. The demographics included in Step 1 were sex, age, marital status, and employment status. Furthermore, the number of children below the age of 5 living at home was included, as this could also be related to getting less sleep. As marital status for most participants was either married (or in a domestic partnership) or single, with relatively few participants falling in the other categories, dummies were created for “married” and “single” only. The same holds for employment status for which dummies were created for “currently employed” and “student.” The compound self-regulation variable was included in Step 2, and bedtime procrastination in Step 3^2^.

The final regression models are reported in Table 3. Step 1 did not yield significant models (*p*s > 0.15). For hours of sleep, fatigue, as well as experienced insufficient sleep, self-regulation showed to be a significant predictor in Step 2 (β = 0.21, –0.36, and –0.38, respectively, *p*s < 0.01), significantly increasing the explained variance (R^2^ change = 0.04, 0.13, and 0.14 respectively; *p*s < 0.01). Adding bedtime procrastination in Step 3 again significantly improved all models; *F_change_*_(1, 167)_ ≥ 20.79, *p*s < 0.001. Notably, the effect of self-regulation was largely reduced in the final models. The total explained variance was 27.1% for hours of sleep, 27.8% for fatigue and 40.9% for experienced insufficient sleep.

**Table 3:** **Final regression models for sleep outcomes**.

	**Hours of sleep**		Δ**R^2^**	**Fatigue**		Δ**R^2^**	**Insufficient sleep**		Δ**R^2^**
	β	***p***		β	***p***		β	***p***	
**STEP 1**									
Sex	0.05	0.46	0.04	−0.03	0.69	0.06	−0.09	0.16	0.04
Age	0.09	0.22		−0.09	0.26		−0.11	0.13	
Marital status^a^									
Married	0.12	0.32		−0.11	0.37		−0.01	0.91	
Single	0.18	0.16		−0.08	0.57		−0.07	0.52	
Employment^b^									
Employed	−0.09	0.19		−0.17	0.02		−0.02	0.71	
Student	−0.01	0.90		−0.02	0.75		−0.02	0.74	
Young children	−0.06	0.45		−0.02	0.79		0.06	0.34	
**STEP 2**									
Self-regulation	−0.06	0.46	0.04	−0.18	0.02	0.13	−0.09	0.23	0.14
**STEP 3**									
Bedtime procrastination	−0.52	<0.001	0.19	0.36	<0.001	0.09	0.56	<0.001	0.23

a Marital status was recoded into two dummy variables: married (1= married, 0 = not married) and single (1 = single, 0 = not single).

b Employment was recoded into two dummy variables: employed (1 = employed, 0 = not employed) and student (1 = student, 0 = not a student).

## Discussion

The present aim was to introduce bedtime procrastination as a novel area of procrastination in the domain of health behavior. It has been known that procrastinators experience problems in various domains, although particular attention has been paid to academic procrastination. Given that insufficient sleep is related to severe outcomes including health problems (Buxton and Marcelli, 2010), our novel findings that procrastinators tend to delay their bedtimes as well are particularly relevant.

Our results allow for important initial conclusions and implications regarding bedtime procrastination as a novel construct in the procrastination literature. First, we found that bedtime procrastination was associated with self-regulation as well as general procrastination, endorsing its position as a form of procrastination. Second, bedtime procrastination was indeed related to general reports of insufficient sleep, above and beyond demographics and self-regulation. In view of the high numbers of participants reporting to get insufficient sleep in an average week, bedtime procrastination further proves its relevance as a construct that deserves future attention in the literature.

The current study is the first to present bedtime procrastination as a possible cause for insufficient sleep. As insufficient sleep is increasingly recognized as causing problems related to mental and physical well-being, it is important to further our understanding of contributing phenomena. In that sense, the current study adds to other sleep research highlighting the alarmingly high rates of people experiencing insufficient sleep (e.g., Moore and Meltzer, 2008; Krueger and Friedman, 2009; Gradisar et al., 2011). Besides the clear practical relevance of studying bedtime procrastination, the phenomenon is interesting from a theoretical point of view as well. For one, bedtime procrastination is a problem that is particularly likely to occur in a state where people have little mental energy, or self-control strength, because the decision to go to bed is inherently made at the end of the day when self-control is typically weaker (Baumeister, 2002). This makes it a special case in comparison to other procrastination problems that may also come about more strongly in states of low self-control but are related to behaviors that can in principle be executed any time of day (e.g., studying) rather than being restrained to the evening. The low self-control circumstances within which bedtime procrastination typically occurs particularly call for efficient strategies that do not rely on willpower to deal with the problem. One suggestion in this regard will be discussed below with our directions for future research.

Another interesting aspect of bedtime procrastination is that, while procrastination typically involves voluntarily delaying aversive tasks (Steel, 2007), going to bed is generally not considered aversive. Instead, we speculate that it is not so much a matter of not wanting to sleep, but rather of not wanting to quit other activities. Interestingly, with the development of electrical devices and the 24/7 entertainment industry, people may be facing many more distractions now compared to several decades ago. Dealing with these distractions might be a plausible cause of bedtime procrastination, where procrastinators experience greater difficulties adhering to their planned bedtime compared to non-procrastinators. Accordingly, bedtime procrastination may be a relatively modern phenomenon, making research on this topic quite timely as well.

Three issues have to be kept in mind when interpreting the current results. The first concerns the advantages and limitations of Amazon Mechanical Turk. To begin with, the use of community samples has obvious advantages over the typically used student samples: especially in the case of bedtime procrastination, students—who tend to have distorted sleeping schedules (Lund et al., 2010)—would not be representative of the general population. Moreover, MTurk samples are generally more diverse than standard internet samples (Buhrmester et al., 2011), which was also shown in the current study which included people from different ethnic backgrounds and with different education levels. At the same time, we acknowledge that the sample is self-selected (i.e., participants themselves pick the studies they want to take part in) and in that sense not fully representative of the general population. For the current purpose, however, we have no reason to believe that the data are in any way invalid or unreliable, as supported by the high internal consistencies of the questionnaires used (see also Buhrmester et al., 2011).

The second issue, a conceptual one, concerns our definition of bedtime procrastination as referring to going to bed rather than sleeping. Of course, there may be a discrepancy between the time someone gets to bed and the time he actually sleeps, which can be due to various reasons. Although these may include some form of procrastination as well (e.g., watching TV in bed), it is less clear to what extent failing to sleep when already in bed is a matter of voluntary delay. In contrast to going to bed, the intention to sleep cannot always be enacted. Therefore, we restrict our conceptualization of bedtime procrastination to going to bed, thereby still assuming that it is related to the amount of sleep one will get—an assumption that was indeed supported by the current findings.

Finally, the interpretation of the found relationships should be considered with caution, as the cross-sectional nature of our study does not allow for causal inferences. An alternative explanation could be, for example, that the causal relation is reverse. For instance, the less people sleep and/or the more they feel fatigue, the less they can exert self-regulation and go to bed on time, hence show bedtime procrastination. Additionally, a confounding variable may explain the relation between self-regulation, bedtime procrastination and sleep outcomes. In addition, it should be kept in mind that the assessment of bedtime procrastination and sleep duration based on general self-reports is probably not as accurate as assessments using daily measurements and actigraphy.

### Directions for future research

Our conclusion that going to bed late is a procrastination problem suggests that typical self-regulation enhancing strategies could be applied to prevent or reduce insufficient sleep. For example, implementation intentions could be particularly promising in this context as they do not require cognitive resources (Webb and Sheeran, 2007). Although research on the effectiveness of implementation intentions on procrastination behavior is limited, it may be worthwhile to apply this technique to bedtime procrastination. More in general, strategies that do not require effort are expected to be most successful in reducing bedtime procrastination.

Another road for future research would be to address which particular aspects of self-control are related to going to bed late (e.g., the inability to quit fun activities, or problems with “getting started”). Relatedly, it would be interesting to identify possible subtypes of bedtime procrastinators. Intuitively, we would predict that a distinction may exist between “active” and “inactive” types of bedtime procrastinators (cf. Chun Chu and Choi, 2005), where the former go to bed later than intended because they are busy doing other things, whereas the latter do so because they just cannot be bothered going to bed at that moment or linger in front of the TV.

Finally, it is important for future research to investigate the relationships between bedtime procrastination and other factors that are known to be related to bedtimes. A first candidate, for example, would be chronotype (morningness/eveningness). Initial data suggest that chronotype is indeed a significant predictor of going to bed later than intended, whereby self-reported eveningness was related to more bedtime procrastination, while the independent effect of self-regulation also remained significant (Broers, 2014).

In sum, the current paper proposes a novel area in which procrastinators may experience problems by introducing bedtime procrastination as a self-regulation perspective on insufficient sleep. Being surprisingly understudied, we advocate giving greater priority to studies investigating procrastination in the domain of health behavior. Approaching health behavior from a procrastination perspective allows for novel insights and strategies to improve mental and physical well-being.

### Conflict of interest statement

The authors declare that the research was conducted in the absence of any commercial or financial relationships that could be construed as a potential conflict of interest.

## Footnotes

^1^Indeed, for two of the three dependent variables (hours of sleep and experienced insufficient sleep) the correlation with bedtime procrastination was significantly stronger than the correlation with general procrastination; Steiger's *Z* = −4.82 and 4.56, respectively, *p*'s < 0.001. For daytime fatigue the correlation with bedtime procrastination did not differ significantly from the correlation with general procrastination, *p* = 0.14.

^2^We chose to include bedtime procrastination rather than general procrastination as this scale was specifically construed to tap into a specific subtype of procrastination that is related to sleeping behavior and that was thus expected (and found: see Table 2) to be a stronger predictor of sleep outcomes. Indeed, substituting general procrastination for bedtime procrastination in the regression analyses did not yield similar outcomes (despite the fact that general and bedtime procrastination were highly correlated; Table 2), further attesting to the discriminant validity of bedtime procrastination.

## Appendix

### Bedtime procrastination scale

For each of the following statements, please decide whether it applies to you using a scale from 1 *(almost) never* to 5 *(almost) always*.

1. I go to bed later than I had intended.
2. I go to bed early if I have to get up early in the morning (R).
3. If it is time to turn off the lights at night I do it immediately (R).
4. Often I am still doing other things when it is time to go to bed.
5. I easily get distracted by things when I actually would like to go to bed.
6. I do not go to bed on time.
7. I have a regular bedtime which I keep to (R).
8. I want to go to bed on time but I just don't.
9. I can easily stop with my activities when it is time to go to bed (R).
